# Investigating the impact of perceived stress and anxiety on nonspecific low back pain among future health care professionals in Hungary: a cross-sectional study

**DOI:** 10.3389/fpsyg.2025.1463414

**Published:** 2025-05-19

**Authors:** Blanka Bernadett Kasza, Kinga Nákity, Regina Finta, Norbert Pásztor, Takayuki Kurokawa, Mariann Sápi, Andrea Domján

**Affiliations:** ^1^Department of Physiotherapy, Faculty of Health Sciences and Social Studies, University of Szeged, Szeged, Hungary; ^2^Doctoral School of Interdisciplinary Medicine, University of Szeged, Szeged, Hungary; ^3^Department of Obstetrics and Gynecology, University of Szeged, Szeged, Hungary; ^4^Department of Traumatology, University of Szeged, Szeged, Hungary; ^5^Independent Researcher, Szeged, Hungary

**Keywords:** perceived stress, anxiety, education, low-back pain, health sciences students

## Abstract

**Introduction:**

Health-science students frequently experience low back pain (LBP), influenced by stress, academic demands, and sedentary lifestyles. This study investigated associations between pain intensity, subjective disability, psychological factors (perceived stress and anxiety), LBP-related knowledge, daily sitting hours, and weekly sports participation in health-sciences students. It further examines the link between psychological factors and nonspecific LBP among Hungarian health-science students.

**Methods:**

In total, 172 students (155 women, 17 men, age: 20.33 ± 1.47, Body Mass Index (BMI): 22.32 ± 3.64) participated in the study. 111 subjects (103 women, 8 men, age: 20.52 ± 1.50, Body Mass Index (BMI): 22.48 ± 3.66) had low back pain (LBP group), and 61 subjects (52 women, 9 men, age: 19.97 ± 1.34, BMI: 22.01 ± 3.61) did not (non-LBP group). The Visual Analogue Scale (VAS), Oswestry Disability Index (ODI), Perceived Stress Scale (PSS), State-Trait Anxiety Inventory (STAI), daily sitting hours, weekly sports participation, and Low Back Knowledge Questionnaire (LKQ) were assessed and analyzed as LBP-associated factors.

**Results:**

Stress level of the LBP group indicates high perceived stress (PSS: *M* = 27.23, SD = 8.38), STAI in the complete sample showed “moderate anxiety” (STAI-S: *M* = 43.30, SD = 9.75; STAI-T: *M* = 40.33, SD = 11.43) with a significant difference of STAI-T (*p* = 0.003) between groups. In both groups, a significant, strong positive correlation was found between perceived stress and anxiety (LBP STAI-S: rs = 0.67, *p* = 0.000; LBP STAI-T: rs = 0.74, *p* = 0.000; non-LBP STAI-S: rs = 0.66, *p* = 0.000; non-LBP STAI-T: rs = 0.73, *p* = 0.000). trait anxiety showed a statistically significant positive association with pain intensity (*β* = 0.264, SE_β = 0.120; *b* = 0.051, SE_b = 0.023; *p* = 0.031), and ODI also had a significant positive correlation (*β* = 0.731, SE_β = 0.052; *b* = 0.438, SE_b = 0.031; *p* < 0.001). No relationship was found between the sitting hours and the other variables of the study in either group.

**Discussion:**

This study found a significant association between trait anxiety and personal experiences of LBP in Hungarian health science students. No link was found between LBP and daily sitting hours or sports participation. Given the high prevalence of stress and anxiety among future healthcare professionals and their established link to LBP, health science curricula should include comprehensive education on the psychosocial aspects of LBP.

**Clinical trial registration:**

ClinicalTrials.gov, identifier NCT05487729 (04/08/2022).

## 1 Introduction

Low back pain (LBP) is a complex condition with various contributing factors, including psychological, social, and biophysical elements, comorbidities, and pain-processing mechanisms (Takegami et al., 2023). Notably, chronic pain and depression often coexist, making it difficult to determine the causal relationship between LBP and psychological distress (Woo, 2010). Nonspecific LBP, where the exact cause is unclear, is a prevalent musculoskeletal issue (Oliveira et al., 2018). While treatment typically focuses on physical symptoms, research suggests a strong link between perceived stress and LBP, emphasizing the importance of psychological factors (Vinstrup et al., 2020; Tsuboi et al., 2017). Consequently, a biopsychosocial approach that combines physical and psychological interventions is likely more effective in reducing pain and disability than conventional treatment (Kamper et al., 2014). Guidelines recommend that LBP management include education on prognosis, warning signs, and staying active while incorporating reassurance and addressing cognitive and behavioral aspects of pain (Oliveira et al., 2018; Chou et al., 2007).

Current recommendations for LBP education prioritize encouraging activity resumption, framing LBP as a common issue, and promoting positive coping mechanisms, rather than solely focusing on anatomical causes (Vlaeyen and Linton, 2000; Waddell, 1996). Maciel et al. stress the importance of evaluating knowledge acquisition, suggesting that increased knowledge may not directly improve clinical outcomes but can indirectly enhance LBP management, pain-triggering factor recognition, and joint protection (Maciel et al., 2009). Supporting this notion, the Hungarian validation of the Low Back Pain Knowledge Questionnaire (LKQ) found higher scores in participants who completed a 3-month back school program that included exercise and education (Kovács-Babócsay et al., 2019). Therefore, health science students, specifically those prone to developing LBP (Taha et al., 2023; Nyland and Grimmer, 2003; Menzel et al., 2016), should be aware that a complex education on LBP could raise awareness about its likelihood and potential causes and may contribute to its prevention. This, in turn, might ultimately reduce the prevalence of LBP among both future healthcare providers and their patients.

Studies have reported that health sciences students frequently experience LBP during their studies (Vujcic et al., 2018; Smith and Leggat, 2004; AlShayhan and Saadeddin, 2018; Ilic et al., 2021; Falavigna et al., 2011). Risk factors include stress, demanding curricula, prolonged studying, physical inactivity, and a sedentary lifestyle, with college students spending significant time in such activities and facing academic, financial, and social stressors that contribute to anxiety (Taha et al., 2023; Boussaid et al., 2023; Castro et al., 2020; Felez-Nobrega et al., 2020). Globally, health sciences students suffer from high rates of anxiety and depression, mirroring the elevated prevalence of LBP in this population (Moutinho et al., 2017; Wege et al., 2016; Seweryn et al., 2015). Heinen et al. (2017) specifically found that medical students experience elevated levels of perceived stress and emotional distress. Consistent with this, Aktekin et al. (2001) reported a significant increase in State-Trait Anxiety Inventory (STAI) scores among Turkish medical students between year one and year two, indicating a decline in psychological wellbeing.

Although LBP is a common musculoskeletal problem, we found no study that has evaluated the effect of the psychological factors on nonspecific LBP and a one-time multidisciplinary education on LBP-related knowledge in health sciences students in Hungary. This study aims to examine the relationship between psychological factors (perceived stress and anxiety) and nonspecific LBP among Hungarian health science students. It also evaluates the effect of a single multidisciplinary educational intervention on students’ LBP-related knowledge. We hypothesize that perceived stress and anxiety are significantly associated with nonspecific LBP among health science students. We assume that students who report LBP within the past 3 months exhibit higher levels of perceived stress and anxiety compared to those without recent LBP. We anticipate that a single multidisciplinary educational session will significantly improve LBP-related knowledge in both students with and without prior LBP experience. Finally, the study explores whether daily sitting time is significantly related to nonspecific LBP among health science students.

## 2 Materials and methods

This pilot study was performed according to the Declaration of Helsinki and was approved by the Ethics Committee of the University of Szeged, Hungary, and the National Public Health Centre (registration No. 48894-7/2020/EÜIG). The ClinicalTrials.gov identifier: NCT05487729 (04/08/2022), was retrospectively registered. All participants provided their signed informed consent before participating in the program. Our study ran between February and July of 2021.

### 2.1 Participants

Participants were recruited from the faculties of medicine and health sciences of the University of Szeged, Hungary, through the official student administration system (Neptun). They were provided informed consent, which included details about the purpose of the study and the use of personal data and results.

To participate, volunteers had to be medicine and health sciences students aged 19–35 and interested in attending a single educational session on LBP (inclusion criteria). Exclusion criteria included failure to complete the post-education questionnaires, a previous diagnosis of specific LBP or another musculoskeletal disease, an acute illness or infection, or known psychological disorders.

During the screening, 211 volunteers (161 from health sciences, 35 medical students, 6 from dentistry, and 9 from pharmacy) met the inclusion criteria. After the educational intervention and applying the exclusion criteria, data from 172 students were included in the final analysis. This group consisted of 137 health sciences students, 25 medical students, 3 dentistry students, and 7 pharmacy students (155 women and 17 men) aged 20.33 ± 1.47 years and a BMI of 22.32 ± 3.64 (Table 1).

**Table 1:** Characteristics of participants.

Characteristics	All participants (*n* = 172)	LBP group (*n* = 111)	Non-LBP group (*n* = 61)	Difference
Gender (n)				
Men	17 (9.88%)	8 (7.20%)	9 (14.75%)	
Women	155 (90.11%)	103 (92.79%)	52 (85.24%)	
Age (years, mean ± SD)	20.33 ± 1.47	20.52 ± 1.50	19.97 ± 1.34	–
BMI (mean ± SD)	22.32 ± 3.64	22.48 ± 3.66	22.01 ± 3.61	–

### 2.2 Outcome measures

Before the educational intervention, participants completed an online, self-administered, structured, closed-ended, multi-item survey via Google Forms® to gather information on their demographics, weekly sports participation in hours and type of sports, average daily sitting hours over a week, and any previous diagnoses of specific LBP, other musculoskeletal diseases, acute illnesses or infections, and known psychological disorders. The intensity of LBP over the past 3 months was evaluated with a digital-based Visual Analog Scale (VAS) in which participants rated pain intensity on a scale from 0 (no pain) to 10 (worst pain) (Delgado et al., 2018).

Based on the VAS scores, participants were assigned either to the group with LBP or without (non-LBP). Only those who marked zero on the VAS scale were assigned to the non-LBP group; other subjects were allocated to the LBP group. Thus, 61 subjects (52 women and 9 men, BMI 22.01 ± 3.61) were in the non-LBP group, while in the LBP group, there were 111 subjects (103 women and 8 men, BMI 22.482 ± 3.66). Participants also filled out the Hungarian-validated versions of the questionnaires listed below using Google Forms®. The LKQ was filled out before and after the educational intervention.

The Oswestry Disability Questionnaire (ODI) was used (Fairbank et al., 1980) for the evaluation of back pain and patients’ self-reported permanent functional disability. The ODI is a self-administered questionnaire, the scores of which are associated with the degree of disability, ranging from minimal to bedbound. According to Valasek et al. (2013), the Hungarian translation of ODI is a valid and reliable assessment tool for patients with LBP, with a Cronbach’s alpha of 0.890. Scores were interpreted according to the standard disability categories (scores from 0 to 20% indicate minimal disability; 20–40%, moderate disability; 40–60%, severe disability; 60–80%, crippled; and 80–100%, bedbound or exaggerating) (Fairbank et al., 1980).

Cohen et al. (1983) assessed perceived stress using the PSS, a self-report scale with two categories: negative perception and positive perception. Higher scores suggest a higher level of perceived stress. Stauder and Konkoly (2006) performed the Hungarian validation of the PSS with a Cronbach’s alpha of 0.88. Since PSS is not a diagnostic tool, there is no standard cutoff score. In our study, the levels of stress were arbitrarily divided into low perceived stress: 0–13, moderate perceived stress: 14–26, and high perceived stress: 27–40. The levels of stress divisions were selected following a similar study by Anandhalakshmi et al. (2016).

For the measurement of trait and state anxiety, subjects had to complete the State-Trait Anxiety Inventory (STAI-T, STAI-S) by Spielberger (1983) and Spielberger and Sydeman (1994). This questionnaire is a psychological inventory consisting of 40 self-report items evaluating state and trait anxiety—STAI scores <40 to indicate no or minimal symptoms and ≥40 to indicate the presence of moderate or severe, clinically significant symptoms (Spielberger and Sydeman, 1994). However, according to Van Dam et al. (2013), a cut-off score ≥40 indicates a need to screen for potential anxiety disorders, while a score ≥43 presents clinically significant anxiety.

To minimize the influence of exam-related stress, the STAI-T and STAI-S questionnaires were administered when stress levels are generally lower for university students, as recommended by previous research (Strack and Esteves, 2015). The Hungarian validation of the questionnaire was performed by Sipos (1978); Sipos et al. (1998), with a Cronbach’s alpha of 0.93.

Students’ knowledge of LBP was specifically measured using the LKQ developed by Maciel et al. (2009). The Hungarian version of this tool was validated by Kovács-Babócsay et al. (2019), and the Cronbach’s alpha was 0.894. This questionnaire comprises 16 items, focusing on spinal anatomy, biomechanics, mechanisms of spinal diseases, the prevention and treatment of spinal diseases, and rehabilitation, categorized into the following groups: general aspects, concepts, and treatment. The score ranges from 0 to 24 points, with a higher score denoting a better knowledge of LBP (Maciel et al., 2009). Participants were instructed to answer the questions independently, relying solely on their knowledge and avoiding the use of external resources. If they were uncertain about an answer, they were advised to select “I don’t know.”

### 2.3 Education and follow-up

After completing the initial questionnaires, participants attended a one-hour educational session on LBP. We provided a single, standardized one-hour education session for all participants in the same setting. The PowerPoint presentation was comprehensive, covering the definition, prevalence, symptoms, and different types of LBP, and strategies for achieving symptom relief. It also discussed prevention techniques, emphasizing the importance of early intervention, and outlined treatment options tailored to the specific type of low back pain in line with the latest scientific guidelines. Additionally, the presentation highlighted key symptoms that warrant consultation with a specialist and examined the role of psychological factors such as stress and anxiety. Two experienced physiotherapists with strong university-level teaching backgrounds and active practice in the field delivered the session. Participants were encouraged to ask questions throughout the session. Following the presentation, participants completed the questionnaires again to assess changes in their knowledge and attitudes about LBP.

### 2.4 Data analysis

Power analysis was conducted using G*Power (Version 3.1.9.2) (G*Power©, University of Dusseldorf, Germany), yielding a minimum sample size of 146 based on PSS and STAI scores (effect size *d* = 0.5, *α* = 0.05, power = 0.9). Statistical analyses were performed using Statistica (version 13.5.017). Data were summarized in descriptive statistics as means and standard deviations. The compliance of the data with the normal distribution probability was analyzed via the Shapiro–Wilk’s W test. It showed that the variables of the study were continuous; daily sitting hours (over a week) and weekly sports participation (in hours) data were normally distributed, but LKQ, VAS, ODI, PSS, STAI-T, and STAI-S data were not. The intensity of physical activity was categorized based on the metabolic equivalent (MET) values of common physical activities, following the classification proposed by Haskell et al. (2007). Three categories were applied: light (<3.0 METs), moderate (3.0–6.0 METs), and vigorous (>6.0 METs). Participants who reported no regular sports participation were assigned to the light-intensity category, as this reflects the level of daily life activities.

Daily sitting hours over a week and weekly sports participation data intergroup differences were evaluated through the independent t-test. Because the data was skewed for LKQ, VAS, ODI, PSS, STAI-T, and STAI-S data, intergroup differences were evaluated through the Wilcoxon-Mann–Whitney U test. The Wilcoxon matched-pairs signed-rank test was used to compare intragroup changes for before and after LKQ data. To adjust for multiple comparisons, we re-ran the Wilcoxon signed-rank test with a Bonferroni correction. A Bonferroni-adjusted *α* of 0.0031 was therefore used to define statistical significance. Results with uncorrected *p*-values below 0.05 but above the Bonferroni threshold were reported as nominally significant. For effect sizes, Hedges’ *g* was calculated for the independent t-test, Cohen’s *d* was calculated for the Wilcoxon-Mann–Whitney U test, and for the Wilcoxon matched-pairs signed-rank test.

Multiple linear regression analysis was conducted to examine the relationship between pain intensity (dependent variable: VAS score) and the following independent variables: subjective disability (ODI), state and trait anxiety (STAI-S, STAI-T), perceived stress (PSS), weekly sports participation, and average daily sitting hours. We used standardized residuals with a threshold of ±2 to identify potential outliers. After excluding eight such cases, diagnostic plots of the residuals indicated no violations of homoscedasticity. Furthermore, variance inflation factor (VIF) values for all predictors remained below the conventional cutoff of 5, suggesting that multicollinearity was not a concern.

For Spearman’s rank correlation, the standard thresholds were used (*ρ* = 1—Perfect positive monotonic correlation, 1 > *ρ* ≥ 0.8—Strong positive monotonic correlation, 0.8 > *ρ* ≥ 0.4—Moderate positive monotonic correlation, 0.4 > *ρ* > 0—Weak positive monotonic correlation, *ρ* = 0—No correlation). Statistical significance was set at *p* < 0.05.

## 3 Results

There was no statistically significant difference between LBP and non-LBP groups in age and BMI (Table 1).

Statistically significant differences were observed in pain, subjective disability, and trait anxiety (*p* < 0.01) (Table 2).

**Table 2:** Study variables.

Variables (mean ± SD)	All participants (*n* = 172)	LBP group (*n* = 111)	Non-LBP group (*n* = 61)	Significant difference	Effect size
Weekly sports participation (in hours)	3.38 ± 2.64	3.39 ± 2.63	3.38 ± 2.67	*p* = 0.98	*g* = 0.00
Daily sitting hours (over a week)	6.45 ± 2.26	6.65 ± 2.07	6.08 ± 2.52	*p* = 0.10	*g* = 0.23
Pain (VAS, 3 months)	–	2.89 ± 1.9	0	^**^p = 0.000	*d* = 0.83
Subjective disability (ODI score)	2.59 ± 3.15	3.77 ± 3.29	0.43 ± 0.99	^**^p = 0.000	*d* = 0.87
LKQ before education scores	17.52 ± 3.05	17.54 ± 2.99	17.47 ± 3.17	*p* = 0.94	*d* = 0.50
LKQ after education scores	19.09 ± 2.47	19.14 ± 2.33	19.01 ± 2.73	*p* = 0.80	*d* = 0.50
Perceived Stress (PSS score)	26.43 ± 8.57	27.23 ± 8.38	24.97 ± 8.78	*p* = 0.09	*d* = 0.57
Anxiety (the State-Trait Anxiety Inventory score)					
STAI-S	43.30 ± 9.75	44.68 ± 9.26	40.80 ± 10.20	*p* = 0.05	*d* = 0.34
STAI-T	40.33 ± 11.43	42.00 ± 11.15	37.30 ± 11.41	^**^p = 0.003	*d* = 0.63

** Difference is significant at *p* ≤ 0.01 level.

*g* is Hedges’ *g* for the independent t-test effect size.

*d* is Cohen’s *d* for Wilcoxon-Mann–Whitney U Test effect size.

The PSS score for all participants indicated moderate stress (Table 2).

Although the stress levels between the two groups did not differ statistically significantly (*p* = 0.06) (see Table 2), the LBP group exhibited higher perceived stress levels compared to the moderate stress levels of the non-LBP participants.

The State-Trait Anxiety Inventory in the complete sample showed “moderate or high anxiety” (Table 2). Furthermore, the state anxiety score of the non-LBP group (46%) and trait anxiety (54%) of non-LBP and state anxiety score of the LBP group (46%) and trait anxiety of the LBP group (60%) exceeding the cut-off score of 40 indicates a need to screen for potential anxiety disorders. Moreover, state anxiety of the LBP group (39%) and trait anxiety of LBP (53%) were found to be high (≥43), indicating clinically significant anxiety, while state anxiety of the non-LBP group (38%) and trait anxiety of the non-LBP group (51%) were found to be high (≥43), as presenting clinically significant anxiety.

There was a statistically significant difference (*p* = 0.003) in trait anxiety level between the LBP and non-LBP groups.

### 3.1 The connection between LBP and psychological factors

The score of VAS in the LBP group indicated mild pain (Table 2). Scores of ODI of both groups belong to the “minimal disability” (Table 2); however, a Mann–Whitney U test indicated a statistically significant difference in subjective disability between the groups (*U* = 858.5, *p* < 0.01).

Results of the Spearman’s correlation indicated a statistically significant (*p* < 0.01), moderate, positive correlation between PSS and anxiety scores in both groups (non-LBP group STAI-T: rs = 0.73, STAI-S: rs = 0.66) (LBP group STAI-T rs = 0.74, STAI-S: rs = 0.67) (Table 3).

**Table 3:** Spearman’s correlation coefficients (rs) of relationships among study variables.

Study variables	VAS	LBP group						Non-LBP group					
		ODI	Weekly sports participation (in hours)	Daily sitting hours (over a week)	PSS	STAI-S	STAI-T	ODI	Weekly sports participation (in hours)	Daily sitting hours (over a week)	PSS	STAI-S	STAI-T
LKQ before	0.06	0.13	0.10	−0.08	−0.26**	−0.21*	−0.17	0.07	0.11	0.02	0.10	−0.06	−0.01
LKQ after	0.18*	0.21*	0.18	−0.04	−0.08	−0.10	−0.03	−0.08	−0.14	0.06	−0.07	−0.25	−0.25*
VAS	1	0.58**	0.10	0.02	0.18*	0.19*	0.27**	–	–	–	–	–	–
ODI	–	1	0.14	−0.07	0.17	0.16	0.19*	1	0.00	−0.10	0.21	0.35**	0.41**
Weekly sports participation (in hours)	–	–	1	−0.02	−0.22*	−0.22*	−0.23*	–	1	−0.11	−0.38**	−0.27*	−0.25*
Daily sitting hours (over a week)	–	–	–	1	0.12	0.13	0.13	–	–	1	0.07	0.09	0.06
PSS	–	–	–	–	1	0.67**	0.74**	–	–	–	1	0.66**	0.73**

Strength of correlations standard thresholds.

*ρ* = 1—Perfect positive monotonic correlation.

1 > *ρ* ≥ 0.8—Strong positive monotonic correlation.

0.8 > *ρ* ≥ 0.4—Moderate positive monotonic correlation.

0.4 > *ρ* > 0—Weak positive monotonic correlation.

*ρ* = 0—No correlation.

^*^Correlation is significant at the *p* ≤ 0.05 level, ^**^Correlation is significant at the *p* ≤ 0.01 level.

A multiple linear regression was conducted to predict LBP intensity (dependent variable: VAS score for the past 3 months) based on psychological factors, including perceived stress (PSS), state anxiety (STAI-S), and trait anxiety (STAI-T). The model was adjusted for subjective disability (ODI score), weekly sports participation, and average daily sitting hours. The regression model was statistically significant (*F* = 41.290, *p* < 0.001), with an *R*^2^ of 0.612. Among the variables, trait anxiety showed a statistically significant positive association with pain intensity (*β* = 0.264, SE_β = 0.120; *b* = 0.051, SE_b = 0.023; *p* = 0.031), and subjective disability (ODI) also had a significant positive correlation (*β* = 0.731, SE_β = 0.052; *b* = 0.438, SE_b = 0.031; *p* < 0.001). In contrast, state anxiety (STAI-S) (*β* = −0.092, SE_β = 0.109; *b* = −0.015, SE_b = 0.018; *p* = 0.390), perceived stress (PSS) (*β* = −0.063, SE_β = 0.075; *b* = −0.013, SE_b = 0.016; *p* = 0.400), weekly sports participation (*β* = 0.010, SE_β = 0.052; *b* = 0.007, SE_b = 0.037; *p* = 0.830), and daily sitting hours (*β* = 0.090, SE_β = 0.050; *b* = 0.076, SE_b = 0.042; *p* = 0.070) did not show statistically significant effects in the model.

Results of Spearman’s correlation indicated that in the non-LBP group, a statistically significant, positive, weak (STAI-S: rs = 0.35, *p* < 0.01), and moderate (STAI-T: rs = 0.41, *p* < 0.01) correlation was found between the ODI scores and anxiety scores. A statistically significant, weak, positive correlation was observed between the ODI and STAI-T scores (rs = 0.19, *p* < 0.05) (Table 3).

### 3.2 LBP-related knowledge

A Mann–Whitney U test indicated no statistically significant difference in LBP-related knowledge between the groups before (*U* = 3,363, *p* = 0.94) or after (*U* = 3,377, *p* = 0.97) education.

LKQ data intragroup analysis showed a statistically significant increase in knowledge in both groups. Wilcoxon signed-rank test output indicated that the after-education scores were statistically significantly higher than before-education scores (LBP: Z = 5.36, *p* < 0.01; non-LBP: *Z* = 3.09, *p* < 0.01).

Following the educational intervention, differences emerged between the two groups. After Bonferroni adjustment for 16 comparisons (adjusted *α* = 0.0031), significant improvements were observed for the LBP group on Questions 4 and 10, and for the non-LBP group on Question 10. Four additional items in the LBP group (Questions 3, 6, 13, 16) and three items in the non-LBP group (Questions 9, 13, 16) yielded *p*-values below 0.05 but did not reach significance under Bonferroni correction, indicating only a trend toward improvement. In summary, while the LBP group showed improvements across concepts, general aspects, and treatment, the non-LBP group’s progress was confined to treatment knowledge. Overall, enhancements in treatment-related knowledge were the most pronounced in both groups compared to improvements in general aspects or conceptual knowledge (see Table 4).

**Table 4:** Means, SD and Wilcoxon matched-pairs signed-rank test for intragroup comparison of LKQ scores.

LKQ	LBP group					Non-LBP group				
	*N*	Before Education Mean ± SD	After Education Mean ± SD	Wilcoxon Z	*p* value/effect size	*N*	Before Education Mean ± SD	After Education Mean ± SD	Wilcoxon Z	*p* value/effect size
Question 1	46	0.57 ± 0.49	0.59 ± 0.49	0.25	*p* = 0.79 *d* = 0.04	21	0.62 ± 0.48	0.60 ± 0.48	0.22	*p* = 0.82 *d* = 0.05
Question 2	22	0.79 ± 0.40	0.86 ± 0.34	1.49	*p* = 0.13 *d* = 0.02	24	0.78 ± 0.40	0.78 ± 0.40	0.01	*p* = 0.98 *d* = 0.00
Question 3	15	0.82 ± 0.37	0.92 ± 0.25	2.49^*^	*p* = 0.01 *d* = 0.48	15	0.78 ± 0.40	0.89 ± 0.30	1.81	*p* = 0.06 *d* = 0.47
Question 4	20	0.79 ± 0.40	0.93 ± 0.24	3.13^**^	*p* = 0.001 *d* = 0.62	12	0.81 ± 0.38	0.90 ± 0.28	1.80	*p* = 0.07 *d* = 0.47
Question 5	5	0.96 ± 0.18	0.99 ± 0.09	1.21	*p* = 0.22 *d* = 0.23	5	0.95 ± 0.20	0.97 ± 0.16	0.40	*p* = 0.68 *d* = 0.10
Question 6	29	1.17 ± 0.37	1.32 ± 0.47	2.74^*^	*p* = 0.005 *d* = 0.54	26	1.18 ± 0.40	1.34 ± 0.48	1.87	*p* = 0.06 *d* = 0.49
Question 7	7	1.96 ± 0.18	1.96 ± 0.23	0.00	*p* = 1.00 *d* < 0	8	1.89 ± 0.34	1.92 ± 0.31	0.70	*p* = 0.48 *d* = 0.18
Question 8	42	1.52 ± 0.55	1.52 ± 0.55	0.00	*p* = 1.00 *d* = 0.00	32	1.47 ± 0.59	1.51 ± 0.56	0.29	*p* = 0.76 *d* = 0.07
Question 9	36	0.18 ± 0.38	0.21 ± 0.41	0.58	*p* = 0.56 *d* = 0.11	17	0.15 ± 0.35	0.29 ± 0.45	2.20^*^	*p* = 0.02 *d* = 0.58
Question 10	79	0.90 ± 0.54	1.50 ± 0.61	5.93^**^	*p* = 0.000 *d* = 1.36	45	0.76 ± 0.52	1.49 ± 0.67	5.16^**^	*p* = 0.000 *d* = 1.76
Question 11	51	1.10 ± 0.66	1.21 ± 0.52	1.37	*p* = 0.16 *d* = 0.26	26	1.13 ± 0.67	1.18 ± 0.59	0.49	*p* = 0.62 *d* = 0.12
Question 12	33	0.78 ± 0.41	0.79 ± 0.40	0.15	*p* = 0.87 *d* = 0.02	19	0.76 ± 0.41	0.68 ± 0.46	1.12	*p* = 0.25 *d* = 0.29
Question 13	46	1.50 ± 0.55	1.64 ± 0.51	2.05^*^	*p* = 0.03 *d* = 0.39	25	1.38 ± 0.66	1.59 ± 0.58	2.24^*^	*p* = 0.02 *d* = 0.60
Question 14	11	0.92 ± 0.25	0.93 ± 0.24	0.26	*p* = 0.78 *d* = 0.05	6	0.90 ± 0.28	0.90 ± 0.28	0.10	*p* = 0.91 *d* = 0.02
Question 15	19	1.78 ± 0.51	1.87 ± 0.33	1.71	*p* = 0.08 *d* = 0.32	20	1.71 ± 0.55	1.78 ± 0.43	0.70	*p* = 0.47 *d* = 0.18
Question 16	7	1.89 ± 0.41	1.97 ± 0.16	2.02^*^	*p* = 0.04 *d* = 0.39	8	1.84 ± 0.46	1.95 ± 0.26	2.24^*^	*p* = 0.02 *d* = 0.59

* Nominally significant difference, *p*-value is between 0.0031 and 0.05.

** Difference is significant at the adjusted *p* ≤ 0.0031 level.

*d* is Cohen’s *d* for Wilcoxon matched-pairs signed-rank test effect size.

### 3.3 Connection of daily sitting hours and weekly sports participation with pain, subjective disability, and psychological factors

Of all participants, 50.58% reported engaging in vigorous-intensity sports. Within the LBP group, 18.91% were classified as engaging in light-intensity physical activity, 36.03% in moderate-intensity, and 45.04% in vigorous-intensity activities. In the non-LBP group, 14.74% reported light, 24.59% moderate, and 60.65% vigorous-intensity physical activity.

There was no statistically significant difference in the hours of sports participation for LBP and non-LBP groups; *t* = 0.01, *p* = 0.98, and in sitting hours for LBP and non-LBP groups; *t* = 1.60, *p* = 0.10 (Table 2).

Results of the Spearman’s correlation indicated a statistically significant, weak, negative correlation between sports participation and psychological factors (LBP group: STAI-S: rs = −0.22, *p* < 0.05; STAI-T: rs = −0.23, *p* < 0.05, and PSS: rs = −0.22, *p* < 0.05; non-LBP group: STAI-S: rs = −0.27, *p* < 0.05; STAI-T: rs = −0.25, *p* < 0.05, and PSS: rs = −0.38, *p* < 0.01). (Table 3).

## 4 Discussion

This study aimed to investigate the correlation between psychological factors and LBP in health sciences students at the University of Szeged in Hungary. The vulnerability of university students to mental health issues and the connection between stress and anxiety are well-documented (Fauzi et al., 2021; Knapstad et al., 2021; Quek et al., 2019; Moreira de Sousa et al., 2018). In the present study, two psychological factors were assessed: perceived stress and anxiety. The main findings indicated that perceived stress levels were moderate in the non-LBP group and high in the LBP group, while both groups demonstrated moderate levels of anxiety. Applying the ≥40 threshold proposed by Van Dam et al. (2013) for identifying possible anxiety disorders, we found that state anxiety scores in the non-LBP group and trait-anxiety scores in the LBP group exceeded this cut-off, indicating a need for formal screening in both subgroups. Furthermore, the LBP group exhibited a state anxiety score of 43 or higher, indicating clinically significant anxiety (Van Dam et al., 2013). Additionally, trait anxiety levels were significantly higher in the LBP group compared to the non-LBP group.

In line with previous findings by Onieva-Zafra et al. (2020) and Herbert (2022), the current study demonstrated an association between perceived stress and anxiety among both groups of health science students. Onieva-Zafra et al. (2020) further highlight that trait anxiety is the strongest predictor of perceived stress. Perceived stress reflects an individual’s subjective evaluation of stress experienced over a specific timeframe (Cohen et al., 1983). State anxiety is typically characterized by heightened vigilance in response to acute stress and anticipation of potential threats (Roozendaal et al., 2008), whereas trait anxiety represents a predisposition to persistent anxiety, increasing susceptibility to state anxiety in challenging situations (Endler and Kocovski, 2001).

Zeng et al. (2023) suggest that perceived stress is a reliable indicator for predicting psychological disorders, such as depression. In addition, Riley et al. (2019) emphasize that elevated stress levels can negatively affect students, contributing to depression and impairing academic performance and overall health. In our study, moderate levels of perceived stress were observed across all participants, aligning with the Hungarian validation of the PSS, which reported similar scores among individuals under 25 and university students. The elevated stress levels in the LBP group might have had negative implications on students’ academic performance and wellbeing. Furthermore, Vinstrup et al. (2020) identify high levels of perceived stress as a significant risk factor for the development of future musculoskeletal disorders. Accordingly, the PSS outcome in the LBP group may underscore the importance of stress in the development of nonspecific LBP among participants.

The current findings support prior research emphasizing the role of psychological factors in developing and maintaining LBP. Brown et al. (2018) propose that anxiety may lead to hypervigilance, triggering neurobiological changes that heighten pain sensitivity. Chronic pain is also frequently associated with anxiety and depressive symptoms (Morley, 2008). Konno and Sekiguchi (2018) highlight the contribution of psychosocial factors to both the onset of LBP and associated disability. Additionally, Bener et al. (2013) report a higher prevalence of anxiety among individuals with LBP than those without. The study not only reaffirms the association between pain, disability, and psychological factors but also emphasizes the specific influence of trait anxiety on LBP severity.

Interestingly, although the non-LBP group did not report experiencing LBP in the past 3 months, they showed minimal levels of disability on the ODI, which positively correlated with anxiety. This suggests that students experiencing anxiety may perceive some level of functional impairment even in the absence of recent LBP. Consequently, anxiety may represent a risk factor for the future development of LBP among students, a notion supported by prior research linking anxiety disorders to the onset of LBP (Arola et al., 2010; Pinheiro et al., 2017).

Another notable finding of the present study was the difference in knowledge improvement related to LBP between groups. Participants with LBP demonstrated significant improvement in six questions across all three main categories of the LKQ (general aspects, concepts, and treatment), while the non-LBP group improved in four items, primarily within the treatment category. Although both groups significantly enhanced their knowledge, the LBP group exhibited a broader gain. This outcome aligns with findings from Hock et al. (2022), where participants with chronic nonspecific LBP (cnsLBP) demonstrated improved cnsLBP knowledge following a back-school program combining exercise and education. Similarly, Kanaan et al. (2023) observe that integrating LBP education into physical therapy improved disease-specific knowledge and supported better treatment outcomes. The present study also confirmed that a single LBP education session can significantly improve participants’ knowledge; however, the observed difference in knowledge gains between the two groups raises further questions. Specifically, it remains to be determined whether the presence of LBP influences the LBP group’s openness and receptivity to disease-specific education. It is plausible that individuals experiencing LBP may be more likely to engage with and effectively use related educational information than those without such experiences. Research suggests that perceiving information as personally relevant enhances engagement, cognitive processing, and retention by increasing motivation, in line with established psychological theories (Johansen et al., 2023). This heightened engagement may be explained by increased cognitive receptivity stemming from personal involvement (Kapitány-Fövény, 2021), which enhances both attention and motivation (Becker et al., 2018) and supports the transfer of personally relevant content into long-term memory (Skinner and Price, 2019).

The current study did not find an association between daily sitting hours, weekly sports participation, and LBP among health sciences students. Although sedentary behavior and physical activity are frequently explored in the context of LBP, findings across the literature remain inconsistent. While some studies have reported that prolonged sitting is associated with an increased risk of LBP (Cho et al., 2012; Subramanian and Arun, 2017; Jiang et al., 2024; Park et al., 2018), others have failed to confirm this relationship (Chen et al., 2009; Lis et al., 2007; da Costa and Vieira, 2009; Bakker et al., 2009; Kwon et al., 2011; Hartvigsen et al., 2000; Roffey et al., 2010). Similarly, our findings showed no significant association between sitting time, weekly sports participation, and pain or disability. Supporting this, Balling et al. (2018) also find no link between self-reported sitting time and subsequent hospital-diagnosed LBP or lumbar disc herniation in a Danish cohort, suggesting that physical activity may be a more meaningful predictor of LBP than sedentary behavior alone.

Moreover, although weekly sports participation was not associated with LBP in our study, we observed a negative relationship between physical activity and psychological factors. This is consistent with previous research demonstrating that regular physical activity contributes to improved mental health and can alleviate symptoms of anxiety (Wegner et al., 2014; Jayakody et al., 2014; Bennett et al., 2015), insomnia (Lopresti et al., 2013), depression (Schuch et al., 2016), stress (Stubbs et al., 2017), and other psychological disorders (Knöchel et al., 2012). Therefore, our findings emphasize the relevance of psychological factors in the context of LBP, suggesting that reducing psychological distress through physical activity may indirectly mitigate the risk of developing LBP.

To our knowledge, this is the first study to investigate the influence of psychological factors on LBP among Hungarian health science students. Our findings have noteworthy implications for students as future healthcare practitioners.

The high prevalence of stress and anxiety among future healthcare professionals, coupled with their established association with LBP, underscores the necessity for health science curricula to incorporate comprehensive education on the psychosocial dimensions of LBP. This approach would equip future practitioners with the knowledge and skills to proactively identify and manage psychosocial risk factors, fostering improved wellbeing for both themselves and their patients.

In the present study, the LKQ was employed as the only validated Hungarian-language instrument available for assessing disease-specific knowledge related to LBP. However, this questionnaire lacks items related to psychological factors influencing LBP. This underscores the necessity for a new, multidisciplinary questionnaire. While the LKQ evaluates general knowledge regarding the anatomy, causes, symptoms, diagnosis, prognosis, concepts, and treatment of LBP, it should be expanded to include items that assess respondents’ awareness of the psychological components contributing to LBP. Such items could help determine whether individuals recognize how psychological states, such as stress, anxiety, and emotional distress, can influence pain perception and whether they are familiar with coping strategies, including breathing exercises and meditation. Educational tools and assessment instruments should incorporate both physical and psychosocial elements to promote a more holistic understanding of LBP. This approach ensures that the condition is not viewed solely as a musculoskeletal issue, but rather as a multidimensional health concern requiring comprehensive and integrated management.

## 5 Limitations

This pilot study has several limitations that should be acknowledged. First, as the data were self-reported, sitting hours and sports participation may have been inaccurately recalled or misreported, potentially leading to over- or underestimation. Second, the cross-sectional design limits the ability to establish causal relationships, as exposure and outcomes were measured simultaneously. Additionally, the study was conducted at a single university, which may limit the generalizability of the findings to all health sciences students in Hungary. Additionally, non-Hungarian speakers were excluded as the questionnaires and assessments used were only available in Hungarian. Future studies should include foreign language students to ensure inclusivity and comprehensive understanding of the student population. Although the gender imbalance in our sample may be viewed as a limitation, it reflects broader national and international trends. Data from the Hungarian Central Statistical Office (n.d.) indicate that women are disproportionately represented among students in medicine, health sciences, and teacher training programs. This pattern is also evident in the workforce, where women comprise approximately 80% of healthcare professionals in Hungary and 78% across the European Union (Eurostat, 2021).

Furthermore, the study did not collect detailed information about sitting behavior, such as posture, breaks, or specific positions, and the intensity of physical activity was not directly measured. Instead, activity intensity was estimated using MET values based on self-reported sport types. These more nuanced aspects of physical behavior may have distinct associations with LBP, as suggested by Heneweer et al. (2011). In conclusion, while the findings contribute to the growing body of knowledge on the psychosocial correlates of LBP, they should be interpreted with caution. Future research should incorporate more comprehensive and objective measures of both physical activity and sedentary behavior to clarify their specific roles in developing and managing LBP.

## 6 Conclusion

Although limited by self-report, cross-sectional design, and single-center sampling, these findings support the adoption of a biopsychosocial framework for preventing and managing LBP in young adults. For health-care professionals, the results highlight three practical implications. Cognitive-behavioral strategies and stress-management techniques should be integrated with exercise-based rehabilitation programs, as this combined approach will likely improve engagement and clinical outcomes. In addition to that, undergraduate curricula should cultivate competencies enabling future practitioners to recognize and address psychosocial risk factors, promoting truly comprehensive, patient-centered care. Overall, effective LBP management extends beyond biomechanical considerations and requires systematic attention to the psychological dimensions of pain.

## Data Availability

The raw data supporting the conclusions of this article will be made available by the authors without undue reservation.
